# Ovarian cancer – American cancer society atlas of clinical oncology

**DOI:** 10.1038/sj.bjc.6601435

**Published:** 2004-01-20

**Authors:** J A Ledermann

**Affiliations:** 1 University College London, UK

This multiauthor book on Ovarian Cancer edited by Ozols is a new addition to the American Cancer Society Atlas of Clinical Oncology series. As the name suggests, pictures and graphic illustrations are prominent and it is this that sets the book apart from other textbooks on ovarian cancer. The first two chapters on pathology and biology use this technique well with numerous clear illustrations of gross pathology and photomicrographs. The text is reasonably comprehensive, although the pathology section lacked information on the pattern of cytokeratin staining which is frequently used to help distinguish ovarian cancers from other malignant epithelial tumours involving the ovary. There is a strong section on genetics that is well illustrated. However, in some sections of the book the quality of graphic reproduction is poor. There is a section on screening and counselling, but it did not include ongoing screening research trials. There are generously illustrated chapters on staging and surgical cytoreduction with a text focusing on staging modalities and some of the controversies surrounding the role of surgery.

Much of the second half of the book is devoted to therapy. Chemotherapy is divided into chapters on primary chemotherapy, developmental chemotherapy, including drug treatments at relapse, and a chapter on high-dose chemotherapy. Recently conducted clinical trials are discussed. References are reasonably up to date, although some references to abstracts presented at the American Society of Clinical Oncology meetings have now been published as final papers. A whole chapter is devoted to high-dose chemotherapy that is now only a relatively small component of developmental chemotherapy. Also, the chapter refers almost exclusively to the North American literature, with little mention of the European data. The chapter on radiation therapy is very detailed but clearly written. It provides a good reference to the field but its emphasis in the book is rather greater than necessary; nowadays radiation is used rather less frequently for the treatment of ovarian cancer. Early stage management and palliative surgery are two clinical areas dealt with in separate chapters. In the former, the table of FIGO staging found in the chapter on staging is repeated, which illustrates some of the difficulties in coordinating a multiauthor text. Biological therapies are likely to become an increasingly important component of ovarian cancer therapy and the chapter on this is well written and illustrated. However, the chief omission is any reference to inhibitors of EGFR or angiogenesis. It is a pity that the chapters on germ cell and sex-cord tumours appear as a ‘bolt on’. They are somewhat incomplete, covering pathology and surgery with virtually nothing on chemotherapeutic management.

The book is supplied with a CD Rom with full text and illustrations (which is a little clumsy to run on an Apple Macintosh). It is a useful accompaniment to the book that is written from a North American perspective drawing mainly on the writing of the world-renowned Fox Chase Cancer Centre, which has extensive experience in the diagnosis and treatment of ovarian cancer. The book is one of many on ovarian cancer. The main criticism is that in some areas it falls in between a comprehensive text and illustrated review. It will appeal mainly to a North American readership and it is a useful addition to the library of any clinical gynaecological oncologist.

